# Comparison of percutaneous cannulated screw fixation and calcium sulfate cement grafting versus minimally invasive sinus tarsi approach and plate fixation for displaced intra-articular calcaneal fractures: a prospective randomized controlled trial

**DOI:** 10.1186/s12891-016-1122-8

**Published:** 2016-07-15

**Authors:** Yongzeng Feng, Xiaolong Shui, Jianshun Wang, Leyi Cai, Yang Yu, Xiaozhou Ying, Jianzhong Kong, Jianjun Hong

**Affiliations:** Department of Orthopaedics Surgery, The Second Affiliated Hospital of Wenzhou Medical University, NO. 109, Xue Yuan West Road, Lucheng District, Wenzhou, Zhejiang Province 325027 China

**Keywords:** Calcaneus, Intra-articular fractures, Minimally invasive, Percutaneous fixation, Sinus tarsi approach, Randomized controlled trial

## Abstract

**Background:**

The management of displaced intra-articular calcaneal fractures (DIACFs) remains challenging and controversial. A prospective randomized controlled trial was conducted to compare percutaneous reduction, cannulated screw fixation and calcium sulfate cement (PR+CSC) grafting with minimally invasive sinus tarsi approach and plate fixation (MISTA) for treatment of DIACFs.

**Methods:**

Ultimately, 80 patients with a DIACFs were randomly allocated to receive either PR+CSC (*N* = 42) or MISTA (*N* = 38). Functional outcomes were evaluated using the American Orthopaedic Foot and Ankle Society (AOFAS) hindfoot scores. Radiological results were assessed using plain radiographs and computed tomography (CT) scans, and postoperative wound-related complications were also recorded.

**Results:**

The average time from initial injury to operation and the average operation time in the PR+CSC group were both significantly shorter than those in the MISTA group (*p* < 0.05). There were significantly fewer complications in the PR+CSC group than those in the MISTA group (7.1 % vs 28.9 %, *p* < 0.001). The calcaneal width immediate postoperatively and at the final follow-up in the MISTA group were obviously improved compared to those in the PR+CSC group (*p* < 0.001). The variables of sagittal motion and hindfoot motion of the AOFAS scoring system in the PR+CSC group were significantly higher than those in the MISTA group (*p* < 0.05). The good and excellent results in the two groups were comparable for Sanders Type-II calcaneal fractures, but the good to excellent rate in the MISTA group was significantly higher for Sanders Type-III fractures (*p* < 0.05).

**Conclusion:**

The clinical outcomes are comparable between the two minimally invasive techniques in the treatment of Sanders Type-II DIACFs. The PR+CSC grafting is superior to the MISTA in terms of the average time between initial injury and operation, operation time, wound-related complications and subtalar joint activity. However, the MISTA has its own advantages in improving the calcaneal width, providing a more clear visualization and accurate reduction of the articular surface, especially for Sanders Type-III DIACFs.

**Trial registration:**

ChiCTRIOR16008512. 21 May 2016.

## Background

The management of displaced intra-articular calcaneal fractures (DIACFs) remains challenging and controversial [1]. Open reduction and internal fixation (ORIF) through an extensile lateral approach has been widely accepted and established as a standard treatment for DIACFs [2, 3]. However, a fairly high wound-related complication rate has been reported with this approach, including wound edge necrosis, dehiscence, hematoma, infection and injury to the sural nerve [4–7].

In an attempt to lower the complication rate, various minimally invasive techniques have recently been introduced, including external fixation, percutaneous fixation, arthroscopically assisted fixation, and minimal incision techniques via medial, modified lateral (such as the sinus tarsi approach), longitudinal, or combined approaches [8–14]. These techniques have been reported as effective in minimizing soft tissue trauma, thereby reducing the incidence of wound-related complications.

As one of the most popular and effective minimally invasive techniques, the sinus tarsi approach not only can fully expose the posterior facet and the anterolateral fragment, but it also significantly reduces the incidence of postoperative wound complications [11, 12, 15]. In order to further shorten the operative time and decrease the wound complication rate, we introduced, in 2006, percutaneous reduction, cannulated screw fixation and calcium sulfate cement (CSC) grafting. A preliminary clinical study carried out by our team found that compared with the traditional L-shaped extensile lateral approach, this new technique allowed earlier weight-bearing, reduced subtalar joint stiffness and a reduced wound complication rate, while improving the patients’ satisfaction [16]. The purpose of the present study was to compare the functional and radiological outcomes and complications of our percutaneous reduction, cannulated screw fixation and CSC grafting with those of the minimally invasive sinus tarsi approach and plate fixation for treatment of DIACFs.

## Methods

### Subjects

Patients with a Sanders Type-II or Type-III calcaneal fracture, who were the consecutive candidates for surgical treatment at our hospital from January 2009 to December 2011, were randomly allocated to either the percutaneous reduction, cannulated screw fixation and CSC grafting (the PR+CSC group) or the minimally invasive sinus tarsi approach and plate fixation (the MISTA group). The patients were randomly divided into the two groups using coin tossing method. Three senior surgeons were randomly assigned to each group to perform the surgeries using either surgical method.

The inclusion criteria were: 1) unilateral Sanders Type-II or Type-III intra-articular calcaneal fracture; 2) the age was greater than or equal to 18 years old; and 3) closed fracture. The exclusion criteria were: 1) severe medical ailments (severe vascular or neurologic injury, diabetes) or contraindications (known local or systemic infection); 2) Sanders Type-IV, bilateral or open calcaneal fractures; 3) severe and polytraumatic injuries or polytrauma of the ipsilateral lower limb when admitted; and 4) refused to accept the treatment plan. All patients agreed to participate in this clinical trial by signing an informed consent form. The study was approved by the Ethical Board Review of the Second Affiliated Hospital of Wenzhou Medical University (Wenzhou, Zhejiang, China), and was performed in accordance with the ethical standards of the Declaration of Helsinki of 1964.

### Preoperative management

All patients were evaluated using preoperative calcaneal radiographs and computed tomography (CT) scans and two-dimensional reconstruction of the injured foot. The swelling of the hindfoot did not need to subside if the patients were randomly assigned to the PR+CSC group. All surgeries were performed with patients placed in the lateral decubitus position under either epidural or spinal anesthesia. Tourniquets were applied routinely.

### Surgical techniques

**Fig. 1:**
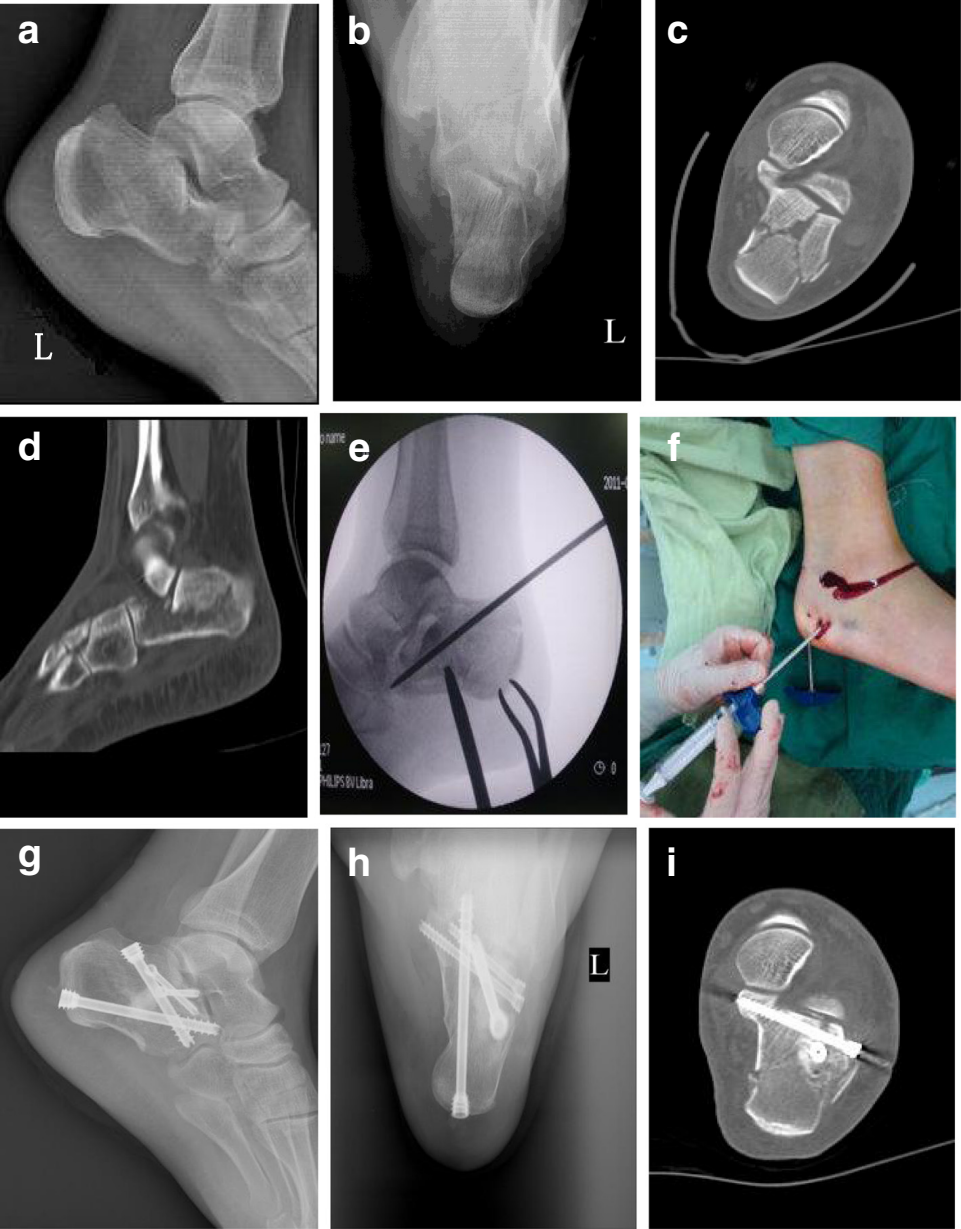
A 42-year-old female patient suffered left lateral displaced intra-articular calcaneal fracture caused by a fall from a height. Preoperative X-ray: lateral view (**a**) and axial view (**b**) showed significantly decreased calcaneal height/length, Bohler angle, Gissanes angle, and significantly increased calcaneal width. A preoperative CT-scan (**c**, **d**) showed a Sanders Type-II. A calcaneal fracture with collapse of the subtalar articular surface. Intraoperative fluoroscopy (**e**) showed the reduction of the subtalar joint surface through two Schanz pins that were used to lever and percutaneously push up the depression of the subtalar joint surface. The CSC was injected into the void of the body (**f**). Postoperative X-ray: lateral view (**g**) and axial view (H) showed calcaneal anatomical reduction. Postoperative CT-scan (**i**) showed the reduction of the articular surface was satisfactory, however, the restoration of calcaneal width was still dissatisfactory

As previously described, the percutaneous reduction, cannulated screw fixation and CSC grafting procedures were briefly summarized as below [16]. First, we crossed the tuberosity with a 6.5-mm Schanz pin via a stab incision to reduce the height and length of the calcaneus. Then, another 6.5-mm Schanz pin was introduced into the fragment with the displaced posterior facet and levered the compressed facet under fluoroscopic guidance. When necessary, especially in Sanders Type-III fractures, another Schanz pin was introduced percutaneously through the lateral cortex of the calcaneus to push up any remaining depressed parts of the subtalar joint surface. Once the Böhler’s angle and articular surface were reduced, two Kirschner wires were inserted from the lateral side to the sustentaculum to sustain the reduced joint surface. Then another two Kirschner wires were introduced from the tuberosity to the anterior part of the calcaneus in different directions to fix the primary and secondary fracture line. After the closed reduction and provisional fixation were performed, the Kirschner wires were replaced by 6.5- and 3.5-mm cannulated screws percutaneously. Finally, the CSC (Wright Medical Technology, Arlington, TN) was then slowly and carefully injected into the bone void in the body created after the reduction under fluoroscopic guidance (Fig. 1).

**Fig. 2:**
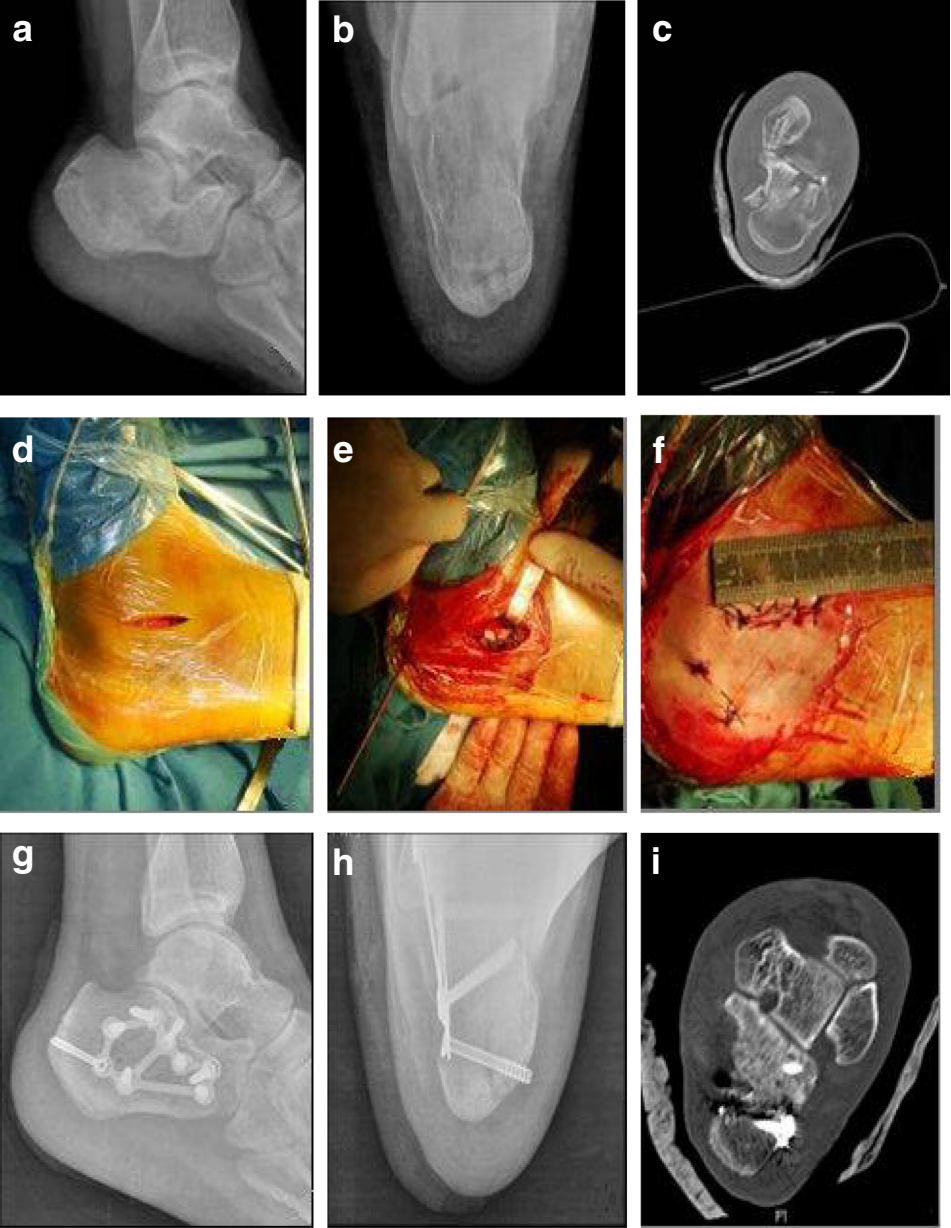
A 38-year-old male patient suffered right lateral displaced intra-articular calcaneal fracture caused by a fall from a height. Preoperative X-ray: lateral view (**a**) and axial view (**b**) showed the calcaneal height/length, Bohler angle, and the Gissanes angle was significantly decreased, and the calcaneal width was significantly increased. Preoperative CT-scan (**c**) showed a Sanders Type-IIIAB calcaneal fracture with collapse of the subtalar articular surface. Intraoperative incision showed a 4.0 cm minimally invasive sinus tarsi approach (**d**), the anatomical plate was placed through the subcutaneous tunnel (**e**), and the incision was sutured (**f**). Postoperative X-ray: lateral view (**g**) and axial view (**h**) showed calcaneal anatomical reduction. Postoperative CT-scan (**i**) showed anatomic reduction of the articular surface and restoration of the calcaneal width

The standard minimally invasive sinus tarsi approach was performed as follows [12]: The front incision was directed toward the lateral wall bone of the anterior process of the calcaneus. Using sharp dissection, this incision was made down to the posterior subtalar joint capsule. After moderate exposure of the lateral wall of the calcaneus, we elevated the subtalar articular surface with a dedicated elevator inserted into the fracture and then a 3.5 mm Steinmann pin was inserted into the calcaneus tuberosity followed by longitudinally extending it to the posterior articular surface to correct the Böhler angle. After reduction, the Steinmann pin was driven into the anterior process of the calcaneus. After the calcaneus length, height and width, and Böhlers and Gissanes angles were recovered satisfactorily under C-arm fluoroscopy, the plate was inserted through the incision. Following the plate insertion, the screws were well fixed through the incision or percutaneously under C-arm fluoroscopy (Fig. 2).

### Postoperative management

All patients in both groups underwent the same postoperative management protocol. The patients were encouraged to do non-weight-bearing exercises including extension and plantar flexion as early as the pain could be endured and use crutch while walking two or three days postoperatively. Partial weight-bearing was permitted at four weeks post-operation and then progressed gradually. Full weight-bearing was not allowed until bony union confirmed on radiographs, which was around three months postoperatively. Each patient received follow-up at 6 weeks, 3, 6, and 12 months post-operation, and then yearly thereafter.

### Clinical evaluation

The functional outcomes were assessed using the American Orthopaedic Foot and Ankle Society (AOFAS) hindfoot scores [17] at the 24-month follow-up. The postoperative wound-related complications were also recorded.

### Radiological evaluation

Lateral and axial radiographs and CT scans were obtained immediately post-operation to assess the reduction of the articular surface and the fracture fixation. Physical examination and lateral and axial radiographs of the injured foot were performed at each follow-up evaluation. The calcaneal anatomical parameters, including Böhlers angle, Gissanes angle, height, width, and length were measured by radiographs or CT scans postoperatively and at the final follow up. Hardware was removed from each patient at 12 months postoperatively, unless the patient had indications for earlier hardware removal.

### Statistical analysis

Statistical analysis was performed with SPSS 17.0 software for Windows. Continuous data with a normal distribution were expressed as the mean ± standard deviation. The Mann-Whitney U test and non-paired t test were used to compare differences continuous variables with non-normal distributions and approximately normally distributed respectively. Categorical data were statistically analyzed by Chi-square test or Fisher’s exact test (*n* < 40 or t < 1). A *p* value of < 0.05 was considered statistically significant.

## Results

**Fig. 3:**
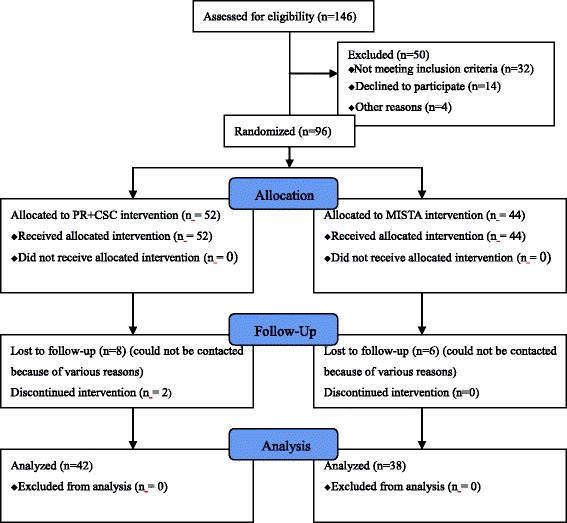
Consolidated Standards of Reporting Trials (CONSORT) 2010 flow diagram depicting fracture allotment in both groups

A total of 146 adult patients were assessed in our study (Fig. 3). Thirty-two patients did not meet the inclusion criteria and were excluded. Eighteen patients were excluded due to severe medical ailments (4 patients) and non-adherence to the treatment plan (14 patients). Therefore, 96 patients with an average age of 40.3 years old (range, 18 to 66 years) including 80 men and 16 women participated in the study. 16 patients were lost during the follow-up. A remaining of 80 patients (66 men and 14 women) were followed-up for 24 months averagely (range, 20 to 29 months).

Forty-two patients were treated with percutaneous reduction, cannulated screw fixation and CSC grafting (PR+CSC group), and 38 patients were treated with the minimally invasive sinus tarsi approach and plate fixation (MISTA group). In the PR+CSC group, there were 34 men and 8 women with a mean age of 39.5 years (range, 18 to 66 years). The affected side of foot included 22 right feet and 20 left feet. According to the Sanders classification, there were 32 Type-II fractures and 10 Type-III fractures. Fall from height (30 fractures) were the primary injury mechanisms, besides motor-vehicle accident (8 fractures), and others (5 fractures). The MISTA group consisted of 32 men and 6 women with an average age of 40.7 years (range, 18 to 65 years). There were 24 right feet injury and 14 left feet injury with 30 Type-II fractures and 8 Type-III fractures. Similarly, the injury mechanisms were also fall from height (30 fractures), motor-vehicle accident (6 fractures), and others (2 fractures; Table 1). There were no significant differences between the two groups regarding sex, age, fracture classification, affected side of foot and injury mechanisms. However, the average time from initial injury to operation was 2.9 days in the PR+CSC group, which was significantly shorter compared with 4.3 days in the MISTA group (*p* < 0.001). The average operation time was also significantly shorter in the PR+CSC group than that in the MISTA group (39.7 min vs 64.2 min, *p* < 0.001; Table 1).

**Table 1:** Comparison of the general characteristics of the two groups

General information	PR+CSC Group	MISTA Group	*P* Value
	*N* = 42	*N* = 38	
Age (yr)	39.5 ± 10.5	40.7 ± 10.3	0.642
Sex			0.702
Male	34	32	
Female	8	6	
Sanders classification			0.768
Type-II	32	30	
Type-III	10	8	
Side of injured			0.330
Right	22	24	
Left	20	14	
Injury mechanism			0.754
Falling accident	30	30	
Traffic accident	8	6	
Other causes	4	2	
Time to operation (days)	2.9 ± 1.1	4.3 ± 1.4	< 0.001
Operation time (min)	39.7 ± 7.6	64.2 ± 8.6	< 0.001

As shown in Table 2, according to the AOFAS scoring system, 22 fractures (52.4 %) were assessed as excellent; 12 (28.6 %) as good; 6 (14.3 %) as fair; and 2 (4.8 %) as poor in the PR+CSC group. In the MISTA group, an excellent, good, fair, or poor result was achieved in 20 (52.6 %), 14 (36.8 %), 3 (7.9 %), and 1 (2.6 %) fracture, respectively. The mean overall AOFAS score was not significantly different between the two groups (PR+CSC group:84.6 ± 6.6 vs MISTA group: 82.5 ± 5.7). Patients in the PR+CSC group had better outcomes on all six variables than patients in the MISTA group. However, there were significant differences between the two groups with regard to the variable of sagittal motion and hindfoot motion in the AOFAS scoring system (*p* < 0.05;).

**Table 2:** The functional outcome according to AOFAS scores for the two groups

	PR+CSC Group	MISTA Group	*P* Value
	(x ± s)	(x ± s)	
Pain	34.0 ± 6.3	33.2 ± 6.2	0.526
Activity limitation	8.4 ± 1.9	8.2 ± 1.9	0.686
Walking surface	3.9 ± 1.5	3.8 ± 1.3	0.784
Gait abnormality	6.0 ± 2.5	5.8 ± 2.4	0.447
Sagittal motion (flexion plus extension)	7.0 ± 1.8	6.0 ± 2.2	0.037
Hindfoot motion (inversion plus eversion)	5.3 ± 1.3	4.5 ± 1.7	0.021
Total	84.6 ± 6.6	82.5 ± 5.7	1.131

Among the patients who achieved good or excellent results, there were 32 Type-II fractures and 2 Type-III fractures in the PR+CSC group. While in the MISTA group, there were 28 Type-II fractures and 6 Type-III fractures. The good to excellent rate of the patients with Sanders Type-II and Sanders Type-(II + III) fractures between the two groups showed no significant differences. However, only for Sanders Type-III fractures, the good to excellent rate in the PR+CSC group was significantly lower than that in the MISTA group (20 vs 75 %; *p* < 0.05; Table 3).

**Table 3:** Good to excellent results in the two groups according to the Sanders classification

Sanders classification	Type-II	Type-III	Type (II+ III)
	no (%)	no (%)	no (%)
PR+CSC Group	32 (100 %)	2 (20 %)	34 (81.0 %)
MISTA Group	28 (93.3 %)	6 (75 %)	34 (89.5 %)
*P* Value	0.230	0.020	0.286

Eight patients developed postoperative wound-healing complications, including superficial surgical site infections (1 patient in the PR+CSC group and 3 patients in the MISTA group), deep surgical site infections (2 patients in the MISTA group), hematoma and wound-edge necrosis (1 patient each in the MISTA group) (Table 4). The superficial surgical site infections and wound hematoma were resolved by dressing changes. The deep surgical site infections, in contrast, involved the hardware, which required hardware removal. The one wound-edge necrosis case was cured by debriding twice and dressing changes. Sural nerve damage was found in two patients in the MISTA group. Musculus peroneus brevis injury was reported in two patients respectively in the two groups, which were sutured immediately intraoperatively and immobilized by plaster for four weeks postoperatively. Overall, the wound-related complication rate was 7.1 % in the PR+CSC group and 28.9 % in the MISTA group, the difference being statistically significant (*p* = 0.010). Two patients had early removal of the implant due to deep surgical site infections, and the other 78 patients had the hardware removed around 12-month post-surgery.

**Table 4:** Comparison of the postoperative wound-related complications in the two groups

Complications	PR+CSC Group	MISTA Group	*P* Value
	no (%)	no (%)	
Wound-healing complications	1 (2.4 %)	7 (18.4 %)	0.024
Superficial infection	1	3	
Deep infection	0	2	
Hematoma	0	1	
Wound-edge necrosis	0	1	
Sural nerve injury	0	2 (5.3 %)	0.222
Musculus peroneus brevis injury	2 (4.7 %)	2 (5.3 %)	1.000
Total	3 (7.1 %)	11 (28.9 %)	0.010

We did not find significant differences in calcaneal Böhlers angle, Gissanes angle, and the height and length between the two groups preoperatively, immediately postoperatively and at the final follow-up. The calcaneal width between the two groups preoperatively also showed no significant difference. However, the calcaneal width immediately postoperatively and at the final follow-up in the MISTA group had obviously improved compared to those in the PR+CSC group (*p* < 0.001; Table 5).

**Table 5:** Radiographic results for the two groups

	PR+CSC Group	MISTA Group	*P* Value
	(x ± s)	(x ± s)	
Böhler angle (deg)			
Preop	2.1 ± 5.5	2.2 ± 7.0	0.921
Immediately Postop	30.3 ± 4.0	30.4 ± 3.3	0.920
Final follow-up	28.6 ± 3.6	29.2 ± 3.5	0.425
Gissanes angle (deg)			
Preop	93.5 ± 7.4	93.3 ± 7.5	0.866
Immediately Postop	120.2 ± 7.2	119.9 ± 6.0	0.830
Final follow-up	117.1 ± 6.8	117.6 ± 6.1	0.724
Calcaneal width (mm)			
Preop	39.8 ± 2.6	39.1 ± 2.5	0.251
Immediately Postop	34.7 ± 2.2	33.0 ± 1.8	< 0.001
Final follow-up	35.3 ± 2.4	33.4 ± 1.9	< 0.001
Calcaneal height (mm)			
Preop	31.4 ± 4.2	30.9 ± 3.6	0.603
Immediately Postop	40.3 ± 5.4	40.6 ± 4.6	0.722
Final follow-up	38.7 ± 2.7	39.3 ± 3.1	0.318
Calcaneal length (mm)			
Preop.	63.5 ± 2.1	63.2 ± 2.8	0.543
Immediately Postop	67.9 ± 4.3	69.1 ± 2.7	0.157
Final follow-up	66.9 ± 3.9	68.2 ± 2.6	0.059

The mean time from surgery to partial weight-bearing was comparable between the PR+CSC group and the MISTA group (6.2 weeks vs 6.0 weeks; *p* < 0.05). At the last follow-up, posttraumatic subtalar arthritis was observed in two patients in the PR+CSC group and one patient in the MISTA group as shown by radiographs.

## Discussion

The optimal management of displaced intra-articular calcaneal fractures remains controversial [1]. An increasing number of studies have shown a trend toward better functional outcomes in the operatively managed groups than those treated non-operatively [18, 19]. However, the high concern about wound-related complications with the extensile lateral L-shaped approach has troubled many orthopedic surgeons [7]. The complication rate after surgical treatment has been reported to range from 11 to 25 % [4–7, 20, 21]. As one of the most widely applied minimally invasive techniques, the sinus tarsi approach has the advantage of direct visualization of the posterior articular facet and fewer wound-related complications [11, 12, 15]. In order to minimize the wound-related complications and explore the optimal treatment for DIACFs, in 2006, we introduced percutaneous reduction, cannulated screw fixation and CSC grafting for treatment of DIACFs. We reported the first clinical data using this technique to treat 90 patients with 90 DIACFs in an article published in 2011 [16]. We found that, compared with ORIF, this minimally invasive technique allowed earlier weight bearing, reduced subtalar joint stiffness, and improved patient satisfaction. To further optimize the minimally invasive percutaneous technique in the treatment of DIACFs, in this study, we aimed to compare the functional outcomes, radiographic results and the postoperative wound-related complications between the minimally invasive percutaneous fixation and the minimally invasive sinus tarsi approach.

The assessment of functional outcomes in our study revealed an overall good to excellent rate of 81.0 % in the PR+CSC group compared with 89.5 % in the MISTA group. The mean AOFAS score was 84.6 in the PR+CSC group, which was higher than that 82.5 in the MISTA group. However, both differences were not statistically significant. There was also no significant difference between the two groups in terms of the good to excellent rate for Sanders Type-II fractures. Wang YM et al. reported percutaneous reduction and Steinman pin fixation minimized complications and achieved functional outcomes comparable to those of the open techniques in patients with Sanders Type-II calcaneal fractures [22]. For Sanders Type-III fractures, however, the MISTA group could achieve better functional outcomes than the PR+CSC group. For this reason, we believed that the minimally invasive sinus tarsi approach was superior to the percutaneous cannulated screw fixation in acquiring a better articular surface reduction, especially for Sanders Type-III fractures. Thus different functional outcomes may be attributed to different qualities of articular reduction. Consistently, Mulcahy et al. also suggessted that a minor residual step-off of the posterior facet could cause a significant load shift within the subtalar joint [23], which might have an adverse effect on functional outcome [24, 25]. Therefore, using the percutaneous reduction and cannulated screw fixation to treat comminuted calcaneal fractures may carry a risk of inadequate reduction of the articular surface.

The results analysis from the partial variables of the AOFAS scoring system suggested that patients in the PR+CSC group suffered from less pain, less activity limitations, less gait abnormality, and less demand on walking surfaces. Meanwhile, they obtained better sagittal motion and hindfoot motion than patients in the MISTA group. However, the difference was only statistically significant for two variables; sagittal motion and hindfoot motion, which suggests, compared with the minimally invasive sinus tarsi approach, that percutaneous reduction and cannulated screw fixation may be more minimally invasive and favor earlier and painless functional exercise.

The radiographic results revealed the significant differences between the two groups in terms of the calcaneal width immediately postoperatively and at the final follow-up. Therefore, the role of the percutaneous reduction and cannulated screw fixation restoring the calcaneal width was weaker than the minimally invasive sinus tarsi approach. Although it is known that the widened calcaneus is the main reason for patients suffering from lateral impingement syndrome [26–28], the difference in the overall functional outcomes between the two groups was not statistically significant. Our data suggested that the reduction of the posterior articular surface might be the most important factor associated with the functional outcome of calcaneal fractures [3, 24, 25].

The wound-healing complication rate was significantly lower in the PR+CSC group than that in the MISTA group (2.4 vs 18.4 %). The minimized disturbance to the blood supply and the soft tissue of the lateral aspect of the calcaneal [29], coupled with the significantly shorter operative time, may have contributed to the lower rate of wound-healing complications in the PR+CSC group. Furthermore, the PR+CSC group had a lower risk of sural nerve injury and musculus peroneus brevis injury compared with the MISTA group, however, the difference was not statistically significant. Although the overall complication rate was found to be quite high in the MISTA group, most of the complications appeared in the early days. The complication rate decreased significantly as our experience improved and the waiting time to operation extended.

There are a few limitations in this study. First, we had a relatively small number of patients and short average follow-up time. Therefore, further investigation with a larger sample size and longer follow-up time is needed to obtain more overall clinical data. In addition, the differences in surgeons’ performances might have decreased the generalization power of this study. Assigning all the patients to only one surgeon would be much beneficial to reduce the variability caused by the surgeon’s performance, but this would be practically impossible in our hospital. Furthermore, the quality of joint surface reduction was not specifically analyzed or compared between the two groups. It needs to be studied further.

## Conclusions

The percutaneous reduction, cannulated screw fixation and CSC grafting for treatment of Sanders Type-II DIACFs can achieve almost equivalent functional outcomes compared with the minimally invasive sinus tarsi approach and plate fixation. The two techniques have their own advantages. The PR+CSC grafting is superior to the MISTA procedure in terms of the average time between initial injury and operation, operation time, wound-related complications and subtalar joint activity. However, the MISTA procedure has its own advantages in improving the calcaneal width, providing clear visualization and more accurate reduction of the articular surface, especially for Sanders Type-III DIACFs. The functional outcomes of Sanders Type-III DIACFs treated by MISTA excelled the PR+CSC grafting.

## Abbreviations

AOFAS, American Orthopaedic Foot and Ankle Society; CONSORT, Consolidated Standards of Reporting Trials; CSC, calcium sulfate cement; CT, computed tomography; DIACFs, displaced intra-articular calcaneal fractures; MISTA, minimally invasive sinus tarsi approach; ORIF, open reduction and internal fixation; PR, percutaneous reduction
